# AI4FoodDB: a database for personalized e-Health nutrition and lifestyle through wearable devices and artificial intelligence

**DOI:** 10.1093/database/baad049

**Published:** 2023-07-18

**Authors:** Sergio Romero-Tapiador, Blanca Lacruz-Pleguezuelos, Ruben Tolosana, Gala Freixer, Roberto Daza, Cristina M Fernández-Díaz, Elena Aguilar-Aguilar, Jorge Fernández-Cabezas, Silvia Cruz-Gil, Susana Molina, Maria Carmen Crespo, Teresa Laguna, Laura Judith Marcos-Zambrano, Ruben Vera-Rodriguez, Julian Fierrez, Ana Ramírez de Molina, Javier Ortega-Garcia, Isabel Espinosa-Salinas, Aythami Morales, Enrique Carrillo de Santa Pau

**Affiliations:** Biometrics and Data Pattern Analytics Laboratory, Universidad Autonoma de Madrid, Calle Francisco Tomas y Valiente, 11, Campus de Cantoblanco, Madrid 28049, Spain; Computational Biology Group, Precision Nutrition and Cancer Research Program, IMDEA Food Institute, CEI UAM+CSIC, Carretera de Cantoblanco, 8, Madrid 28049, Spain; Biometrics and Data Pattern Analytics Laboratory, Universidad Autonoma de Madrid, Calle Francisco Tomas y Valiente, 11, Campus de Cantoblanco, Madrid 28049, Spain; GENYAL Platform on Nutrition and Health, IMDEA Food Institute, CEI UAM+CSIC, Carretera de Cantoblanco, 8, Madrid 28049, Spain; Biometrics and Data Pattern Analytics Laboratory, Universidad Autonoma de Madrid, Calle Francisco Tomas y Valiente, 11, Campus de Cantoblanco, Madrid 28049, Spain; GENYAL Platform on Nutrition and Health, IMDEA Food Institute, CEI UAM+CSIC, Carretera de Cantoblanco, 8, Madrid 28049, Spain; GENYAL Platform on Nutrition and Health, IMDEA Food Institute, CEI UAM+CSIC, Carretera de Cantoblanco, 8, Madrid 28049, Spain; Department of Nursing and Nutrition, Faculty of Biomedical and Health Sciences, Universidad Europea de Madrid, Calle Tajo s/n, Villaviciosa de Odon, Madrid 28670, Spain; GENYAL Platform on Nutrition and Health, IMDEA Food Institute, CEI UAM+CSIC, Carretera de Cantoblanco, 8, Madrid 28049, Spain; Molecular Oncology and Nutritional Genomics of Cancer Group, IMDEA Food Institute, CEI UAM+CSIC, Carretera de Cantoblanco, 8, Madrid 28049, Spain; GENYAL Platform on Nutrition and Health, IMDEA Food Institute, CEI UAM+CSIC, Carretera de Cantoblanco, 8, Madrid 28049, Spain; GENYAL Platform on Nutrition and Health, IMDEA Food Institute, CEI UAM+CSIC, Carretera de Cantoblanco, 8, Madrid 28049, Spain; Computational Biology Group, Precision Nutrition and Cancer Research Program, IMDEA Food Institute, CEI UAM+CSIC, Carretera de Cantoblanco, 8, Madrid 28049, Spain; Computational Biology Group, Precision Nutrition and Cancer Research Program, IMDEA Food Institute, CEI UAM+CSIC, Carretera de Cantoblanco, 8, Madrid 28049, Spain; Biometrics and Data Pattern Analytics Laboratory, Universidad Autonoma de Madrid, Calle Francisco Tomas y Valiente, 11, Campus de Cantoblanco, Madrid 28049, Spain; Biometrics and Data Pattern Analytics Laboratory, Universidad Autonoma de Madrid, Calle Francisco Tomas y Valiente, 11, Campus de Cantoblanco, Madrid 28049, Spain; GENYAL Platform on Nutrition and Health, IMDEA Food Institute, CEI UAM+CSIC, Carretera de Cantoblanco, 8, Madrid 28049, Spain; Biometrics and Data Pattern Analytics Laboratory, Universidad Autonoma de Madrid, Calle Francisco Tomas y Valiente, 11, Campus de Cantoblanco, Madrid 28049, Spain; GENYAL Platform on Nutrition and Health, IMDEA Food Institute, CEI UAM+CSIC, Carretera de Cantoblanco, 8, Madrid 28049, Spain; Biometrics and Data Pattern Analytics Laboratory, Universidad Autonoma de Madrid, Calle Francisco Tomas y Valiente, 11, Campus de Cantoblanco, Madrid 28049, Spain; Computational Biology Group, Precision Nutrition and Cancer Research Program, IMDEA Food Institute, CEI UAM+CSIC, Carretera de Cantoblanco, 8, Madrid 28049, Spain

## Abstract

The increasing prevalence of diet-related diseases calls for an improvement in nutritional advice. Personalized nutrition aims to solve this problem by adapting dietary and lifestyle guidelines to the unique circumstances of each individual. With the latest advances in technology and data science, researchers can now automatically collect and analyze large amounts of data from a variety of sources, including wearable and smart devices. By combining these diverse data, more comprehensive insights of the human body and its diseases can be achieved. However, there are still major challenges to overcome, including the need for more robust data and standardization of methodologies for better subject monitoring and assessment. Here, we present the AI4Food database (AI4FoodDB), which gathers data from a nutritional weight loss intervention monitoring 100 overweight and obese participants during 1 month. Data acquisition involved manual traditional approaches, novel digital methods and the collection of biological samples, obtaining: (i) biological samples at the beginning and the end of the intervention, (ii) anthropometric measurements every 2 weeks, (iii) lifestyle and nutritional questionnaires at two different time points and (iv) continuous digital measurements for 2 weeks. To the best of our knowledge, AI4FoodDB is the first public database that centralizes food images, wearable sensors, validated questionnaires and biological samples from the same intervention. AI4FoodDB thus has immense potential for fostering the advancement of automatic and novel artificial intelligence techniques in the field of personalized care. Moreover, the collected information will yield valuable insights into the relationships between different variables and health outcomes, allowing researchers to generate and test new hypotheses, identify novel biomarkers and digital endpoints, and explore how different lifestyle, biological and digital factors impact health. The aim of this article is to describe the datasets included in AI4FoodDB and to outline the potential that they hold for precision health research.

**Database URL** https://github.com/AI4Food/AI4FoodDB

## Introduction

Precision nutrition has emerged as an essential tool in the prevention and treatment of non-communicable diseases (NCDs). These diseases, in particular those related to dietary imbalances, represent a significant burden on public health: behavioral and dietary habits are among the most relevant risk factors for the development of NCDs, causing 11 million deaths in 2017 (1). For instance, the prevalence of obesity nearly tripled between 1976 and 2016, and it is projected to affect >1900 million adults by 2030 (2). Strategies based on general ‘one-size-fits-all’ dietary recommendations are not enough to prevent these diseases as each individual is defined by a set of unique characteristics such as genetics, gut microbiome, metabolism, bodies, tastes and lifestyles (3). Moreover, personal nutritional requirements are also continuously changing due to many factors like ages, physical conditions, social factors or during circumstances such as pregnancy or illness. This underscores the need for personalized approaches that address an individual’s unique circumstances (4). The relevance of these approaches to address health problems affected by dietary habits has been stated by the ELIXIR Food & Nutrition (F&N) Community (5).

A significant effort has been made to identify biomarkers that explain the variability in patient responses to dietary interventions. This includes genetic polymorphisms present in regions relevant for the metabolism of different molecules, as well as changes in the gut microbiota (GM), i.e. the microbes residing in the digestive tract. For instance, differences in GM composition can be related to the variability in physiological responses after meal intake (6, 7); several studies have identified species in the GM that correlate with nutrient intake reports and blood markers (8, 9), as well as with different diet-related NCDs such as obesity or type 2 diabetes (T2D) (10, 11). Other approaches have focused on the stratification of patients suffering from the same disease. A good example of this is the proposition of the ‘metabolically healthy obesity’ phenotype to describe patients that, despite having a BMI of >30 kg/m^2^, show good cardiovascular health and are at a lower risk of complications such as cardiovascular disease or T2D (12). These are only some examples on how nutritional research can address the personal situation of each subject to tailor nutritional and lifestyle recommendations to their needs.

However, much work still must be done in terms of patient care, follow-up and monitoring. Nutritionist visits traditionally record physical activity and lifestyle data through the use of surveys and validated questionnaires, which are completed during the clinic visit or at home by the patient. These methods are well settled in clinical practice and are widely used in different health-related scenarios, including but not limited to nutrition (13). Usually, they are accompanied by the collection of biological samples in order to measure the presence of different molecules and biomarkers. However, collecting this information is costly and time-consuming, requiring invasive tests in controlled scenarios and burdening both the patient and the clinician. This slows data acquisition and limits the use of these approaches to large cohorts (14). Moreover, variations of parameters such as glycemia or heart rate (HR) throughout the day, which depend not only on meal intake or physical activity but also on the personal circumstances of each patient, are complicated to monitor through these strategies.

As a consequence, e-Health approaches that work toward a shift from paper-based questionnaires to automatic data recording strategies are becoming increasingly common (15). In addition, people’s concerns regarding self-managed health care have increased over the last few years, and the use of health devices (e.g. web-based applications, smartwatches and wearable sensors) has become widespread (16). Consequently, personalized attention via wearable sensors has been recently integrated as a powerful e-Health tool. Vital signs such as HR, body temperature, oxygen saturation (SpO_2_) or respiration rate are now continuously being monitored. These sensors are then integrated into Internet-of-things and smart devices that incorporate technology based on artificial intelligence (AI), responsible for detecting changes in the individual (17). Wearable devices are expected to transform current health care by monitoring the individual continuously in a non-invasive or minimally invasive manner, providing reliable information even in real and uncontrolled scenarios (18).

The use of e-Health and telemedicine approaches to aid patient monitoring in different disease stages, from diagnosis to rehabilitation, has experienced a great increase due to the Coronavirus Disease 2019 (COVID-19) pandemic, in an attempt to alleviate the pressure exerted on national health systems (19). Faced with a large amount of dispersed information, automatic approaches, and specifically, AI-based methods, have offered an effective solution in different fields such as biomedicine and health informatics. Machine learning (ML), a subfield of AI, is today a great solution to handle these vast amounts of information (20, 21). Sleep and physical activity, continuous glucose monitoring (CGM), stress management and HR tracking, among others, are some examples that use ML approaches to detect and prevent some of the most prevalent NCDs (22, 23).

Despite the increasing use of wearable devices in health and nutrition-related fields, current approaches still face many problems. For instance, today’s technology lacks the robustness and user-friendly devices necessary to accurately measure the nutrient intake of each individual (24, 25). Instead, several studies have opted to provide smartphone apps to track participants’ food intake (26): in the work of Lozano-Lozano *et al.* (27), they developed an app to promote lifestyle behavioral changes of breast cancer survivors; others focused on developing smartphone and web-based apps for overweight and obese people to improve their health through weight loss (28, 29).

Several studies have shown that the use of wearable devices can improve both physical and mental conditions of individuals (30). Some approaches aimed to mitigate cardiovascular diseases such as atrial fibrillation, arrhythmia or heart failure (31–33), while others promoted healthy lifestyle behaviors by increasing physical activity (34, 35). Recent studies have shown the effectiveness of using both wearable devices and ML approaches together with other types of health data sources, for instance, electronic health records, metabolic profiles, gut microbiome and diet: some studies investigated how blood glucose levels vary among individuals after consuming standardized meals to combat diabetes (36), while others used CGMs and wearables devices to track food intake and physical activity in order to analyze the impact of glucose deviations during eating or exercising (37–40). Zeevi *et al.* (7) also measured blood parameters, anthropometrics and lifestyle behaviors to demonstrate that personalized nutrition is a key factor to control glycemia.

**Figure 1.**
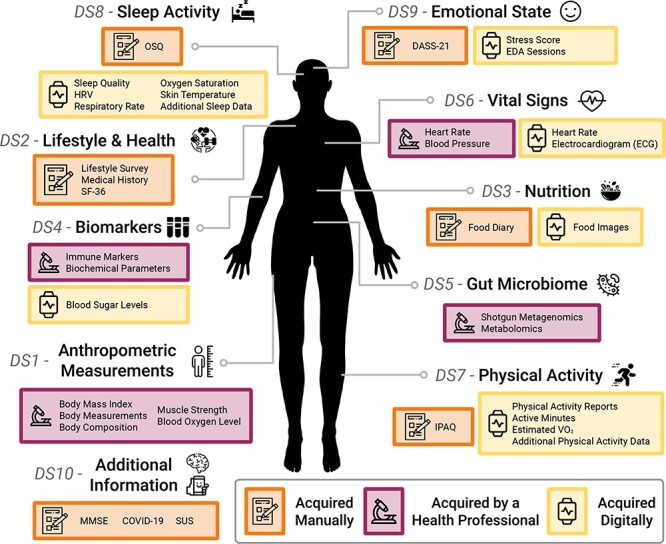
An overview of the AI4FoodDB. The database comprises 10 datasets representing different types of data acquired during the nutritional intervention: Anthropometric Measurements (DS1), Lifestyle and Health (DS2), Nutrition (DS3), Biomarkers (DS4), Gut Microbiome (DS5), Vital Signs (DS6), Physical Activity (DS7), Sleep Activity (DS8), Emotional State (DS9) and Additional Information (DS10). Data acquisition methods are indicated by different colored boxes as well as by icons: manual (depicted with a notebook), clinical (microscope) or digital (smartwatch).

In this context, the AI4Food framework has recruited 100 obese and overweight participants through validated questionnaires, wearable devices, anthropometric measurements and biological samples, with the aim of closely monitoring their lifestyle and health during the course of a 1-month weight loss intervention. Figure 1 provides a graphical description of the different information acquired in the AI4Food framework. Our goal is to work toward a comprehensive human body map or ‘digital twin’ by using the latest advances in technology and the new tools that monitor this biological, physiological and lifestyle information. We expect that the integration of heterogeneous data will allow us to identify complex biomarkers, moving away from nutritional advice based solely on validated questionnaires. Consequently, the AI4Food framework aims to contribute to e-Health and the challenges defined by the ELIXIR F&N Community (5) by developing new AI technologies that allow for automatic and user-friendly, yet accurate, patient follow-up. Here, we present the data collected within this framework, the AI4Food database (AI4FoodDB), comparing the various methods employed to monitor participants and providing examples of how these data can be used. The article is organized as follows. The section ‘AI4FoodDB: acquisition Setup’ describes the design and acquisition setup of the AI4FoodDB database. The section ‘AI4FoodDB: datasets’ details the 10 different datasets defined in AI4FoodDB, representing different types of data acquired during the nutritional intervention. Then, we briefly describe in the section ‘Case study: participant follow-up’ a case study of a single participant from the AI4FoodDB, and finally, we draw up future studies and conclusions in the section ‘Conclusions’. To the best of our knowledge, AI4FoodDB is the first public database that centralizes food images, wearable sensors, validated questionnaires and biological samples from the same intervention. As a result, we believe that this database will enhance the current personalized nutrition benchmark and help to better understand human body behavior in NCDs.

## AI4FoodDB: acquisition setup

The AI4FoodDB is designed to build a comprehensive map of the human being by employing the latest advances in biology, sensing and AI technologies. The proposed database also aims to serve as a benchmark in the fields of nutrition, lifestyle and health informatics. Lifestyle, nutritional and biological data have been gathered using diverse methodologies, including the use of wearable devices, validated questionnaires and biological samples. The acquisition of AI4FoodDB was jointly conducted by the IMDEA Food Institute from Madrid, Spain, and the Biometrics and Data Pattern Analytics Laboratory from Universidad Autonoma de Madrid (UAM), Madrid, Spain.

### Ethical issues

The protocols and methodology used in AI4FoodDB comply with the ethical principles for research involving human subjects laid down in the Declaration of Helsinki (1964) and its modifications. The intervention was approved by the Research Ethics Committee of the IMDEA Food Foundation (reference IMD: PI-052, 5 April 2022). Participants were informed in detail about the different stages of the nutritional intervention both orally and in writing. In addition, participants had to sign an informed consent prior to taking part in the study. This document included a section on the storage of the remaining samples and the collected data according to Spanish legislation (Royal Decree 1716/2011 of 18 November). The dissociation criteria are also applied to the volunteer’s data for anonymization, in compliance with the current Spanish legislation (Organic Law 15/1999 of 13 December, on the protection of Personal Data).

### Participants’ description

AI4FoodDB comprises initially a total of 100 overweight and obese participants. Recruitment was carried out by the Platform for Clinical Trials in Nutrition and Health (GENYAL) at the IMDEA Food Institute. This was done by (i) contacting volunteers from the Platform’s database, (ii) advertising the trial in the IMDEA Food Institute website and social media and (iii) disseminating the information in the Cantoblanco Campus and collaborating entities. Seven participants quit the study before the end of the intervention. Table 1 outlines the specific criteria used for participant selection, including both the inclusion and exclusion criteria. A description of the participants involved in the study, including a comparison between Groups 1 and 2, is shown in Table 2. As shown in the table, the two intervention groups showed no significant differences in anthropometric, HR and biochemical measurements.

**Table 1.** The full inclusion and exclusion criteria considered in the AI4FoodDB nutritional intervention.

Inclusion criteria
‒ Age ≥ 18 years
‒ BMI^1^ between 27–35 kg/m^2^
‒ Able to understand the informed consent
‒ Willing to comply with the study protocol
‒ Able to use wearable devices for data collection
Exclusion criteria
‒ Cognitive impairment or neurological disorders
‒ Ongoing severe disease (e.g. liver and kidney)
‒ Pregnant or lactating
‒ Taking medications for weight loss
‒ Unwilling to be monitored by wearable devices and nutritionist visits
‒ Unwilling to comply with guidelines for healthy weight loss

1 BMI: Body Mass Index.

**Table 2.** A description of the participants included in AI4FoodDB.

	Total (*n* = 100)	Group 1 (*n* = 50)	Group 2 (*n* = 50)	*P*-value	Adjusted *P*-value
Sex (female, %)	69	68	70	0.8	> 0.9
Age (years)	50	51 ± 13	48 ± 13	0.3	> 0.9
BMI^1^ (kg/m^2^)	30.66 ± 3.41	29.96 ± 2.88	31.37 ± 3.76	0.084	> 0.9
% Obese	50	46	54	0.4	> 0.9
Waist/hip ratio	0.97 ± 0.88	0.88 ± 0.09	1.06 ± 1.19	0.6	> 0.9
Basal metabolic rate (kcal)	1,617 ± 292	1,591 ± 314	1,644 ± 269	0.6	> 0.9
Intervention diet (kcal)	2,082 ± 349	2,063 ± 326	2,102 ± 373	0.8	> 0.9
HR (bpm)	72 ± 11	71 ± 11	72 ± 10	0.6	> 0.9
Dyastolic blood pressure (mmHg)	78 ± 10	78 ± 7	78 ± 11	> 0.9	> 0.9
Systolic blood pressure (mmHg)	123 ± 17	123 ± 17	124 ± 17	0.7	> 0.9
Total cholesterol (mg/dl)	200 ± 32	198 ± 30	202 ± 33	0.3	> 0.9
HDL^2^ cholesterol (mg/dl)	59 ± 14	57 ± 14	60 ± 15	0.3	> 0.9
LDL^3^ cholesterol (mg/dl)	120 ± 26	120 ± 24	120 ± 19	> 0.9	> 0.9
Triglycerides (mg/dl)	106 ± 46	102 ± 38	110 ± 53	0.9	> 0.9
Glucose (mg/dl)	82 ± 8	83 ± 8	82 ± 8	0.5	> 0.9
HbA1c^4^ (%)	5.63 ± 0.34	5.63 ± 0.33	5.63 ± 0.35	0.8	> 0.9
Insulin (μUI/ml)	10.5 ± 7.4	9.9 ± 7.2	11.0 ± 7.7	0.5	> 0.9
HOMA-IR^5^	2.18 ± 1.69	2.08 ± 1.65	2.29 ± 1.73	0.6	> 0.9
Adiponectin (μg/ml)	13 ± 8	13 ± 8	13 ± 8	> 0.9	> 0.9

Continuous variables are shown as mean ± SD. Differences between the groups are compared using the *t*-test for normally distributed data (tested with the Shapiro–Wilk test with a significance level at 0.05) and the Wilcoxon rank sum test for non-normal variables. Categorical variables were compared using Pearson’s chi-squared test. All *P*-values were corrected for multiple testing via the Benjamini–Hochberg false discovery rate correction.

^1^ BMI: Body Mass Index ^2^ HDL: High-density lipoprotein ^3^ LDL: Low-density lipoprotein ^4^ HbA1c: Hemoglobin A1C ^5^ HOMA-IR, homeostatic model assessment for insulin resistance.

### Types of data

We consider three types of acquisition depending on how the data were collected: (i) manual, where both experts and participants filled out medical and lifestyle standard questionnaires; (ii) clinical, where a health professional was responsible for data collection, including anthropometric measurements and biological sample collection (blood, saliva, urine and stool) and (iii) digital, where wearable sensors recorded physical activity, biological signal information and blood glucose levels from the participants. Figure 1 provides a graphical representation of the information acquired within the AI4Food framework. The different data collection methods considered in this framework and the resulting available information make AI4FoodDB a unique and complete database for precision nutrition research.

### Acquisition protocol

**Figure 2.**
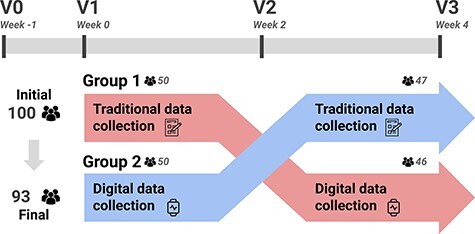
A summary of the acquisition protocol considered in AI4FoodDB. The intervention followed a cross-over design. Participants used a single data collection method for the first 2 weeks (e.g. Group 1 started with traditional data collection) and then switched to the opposite method (in the case of Group 1, digital data collection) for the remaining 2 weeks.

The nutritional intervention lasted 5 weeks, with four different visits as depicted in Figure 2. First, during volunteer recruitment (V0, Week −1), potential participants were informed of the study design, signed the informed consent and were randomly assigned to an intervention group. Then, participants were provided with the information they needed to follow the study. All participants were given nutritional and lifestyle guidelines for healthy weight loss at visit V1 (Week 0), reducing ~500 kcal out of their total metabolic rate during 4 weeks. A varied, balanced diet was designed for each participant according to their specific needs. Moreover, participants were informed about the data that would be collected by digital sensors and were provided with instructions on their use and installation.

As shown in Figure 2, the intervention followed a cross-over design. This means that, on visits V1–V3 (Weeks 0–4), all participants used both collection methods. However, the order in which these methods were used was different orders according to the intervention group. Participants in Group 1 started with the traditional/manual data collection between visits V1 and V2 (Week 0 and Week 2) and then switched to the digital collection for visits V2 and V3 (Week 2 and Week 4), while those in Group 2 did the opposite, starting with the digital data collection and then switching to the traditional/manual collection. Regarding the clinical data, biological samples including blood, saliva, urine and stool samples were also collected at visits V1 (Week 0) and V3 (Week 4).

### Wearable devices and software

We provide next a description of the wearable devices and software considered in AI4FoodDB for digital acquisition:

- FitBit Sense Smartwatch (https://www.fitbit.com/): this is a commercial device designed by the FitBit company. The device, which can be used on any wrist, is very light (~46 g, 1.26 Oz), and the battery lasts ~4–6 days. The smartwatch has also Bluetooth connectivity to synchronize with the FitBit app, available for Android and iOS smartphones. The device incorporates the latest sensorial advances and algorithms to monitor and control the health care of the individual: (i) optical HR sensor to detect HR activity, variability or even oxygen saturation during sleep activity; (ii) electrodermal activity (EDA) sensor to detect stress levels; (iii) gyroscope and accelerometer inertial sensors; (iv) skin temperature sensor to monitor changes; (v) airflow measurement sensor and microphone to detect the respiratory rate while sleeping; (vi) electrocardiogram (ECG) sensor to record heart electricity signals; (vii) Global Positioning System (GPS) and Global'naya Navigatsionnaya Sputnikovaya Sistema (GLONASS) sensors for geolocalization and physical activity detection and (viii) algorithms to detect sleep quality and stages.
- Freestyle Libre 2 (https://www.freestyle.abbott/): this is a glucometer sensor designed by the Abbot Laboratories company. The device is even smaller and lighter than the smartwatch (~5 grams, 0.17 Oz), which is placed on the back of the upper arm and lasts up to 14 days. The glucometer is connected to a smartphone via Near Field Communication (NFC) technology using the FreeStyle LibreLink app (available for Android and iOS smartphones) and monitors the blood sugar levels every 15 min.
- Food Logging Web Platform: participants were asked to take pictures from their personal smartphones (provided if needed) of all the consumed food and drinks (except water) during the trial. For that purpose, we created a mobile-friendly web platform that stored in the cloud all food images with a timestamp of the moment when the picture was taken. Participants simply had to log in to the platform and then upload the images at any time of the day. The platform was developed using the Flask framework, coded in Python. The image database was stored in Google Firebase, providing a fast and secure platform for the participants.

In addition, participants were provided with guidelines for using wearable devices and the mobile web platform properly. Regarding the smartwatch, participants were instructed to wear the device at all times, including during sleep. Participants utilized the FitBit app on their personal smartphones to synchronize data between the smartwatch and FitBit servers. While the smartwatch is capable of storing data for up to 1 week, we requested that participants synchronize their devices on a daily basis to monitor their activity. Moreover, they had to perform a daily ECG and EDA test to monitor heart and stress health, respectively.

For the glucose monitoring device, participants were instructed to scan the sensor with a compatible NFC-enabled smartphone at least every 8 h, which amounts to approximately three times a day. This was to ensure that as much information as possible was captured, as the device only stores 8 h of data. After scanning the data with the smartphone, it was automatically transmitted to the Abbot Laboratories servers via the FreeStyle LibreLink app.

Additionally, participants were requested to take pictures of their food and drinks consumed throughout the day and upload them to the food logging web platform. The collected participant data were manually monitored and reviewed daily for all wearable devices. Finally, all data were downloaded from their respective servers and subjected to preprocessing following a data cleaning procedure.

## AI4FoodDB: datasets

We provide next a description of the 10 distinct datasets defined in AI4FoodDB, representing different types of data acquired during the nutritional intervention, as depicted in Figure 1.

### Dataset 1: anthropometric measurements

During the physical examination at V0, V2 and V3 visits, the following anthropometric measurements were recorded:

- Body mass index (BMI): body weight (kg) and height (cm) were measured and used to estimate the BMI as the body weight divided by the squared height (kg/m^2^).
- Body measurements: waist and hip circumference were measured in cm.
- Body composition: fat mass percentage, muscle mass percentage and visceral fat level were measured via bioelectrical impedance analysis.

As a result, a total of nine variables were collected for each participant at three different time points. Additionally, a measurement of muscle strength was recorded using a digital hand dynamometer at V0 and V3, providing five more variables for each participant, and blood oxygen levels, represented by two variables, were measured using an oximeter during V2.

### Dataset 2: lifestyle and health

At V0, participants completed a lifestyle survey that includes information on appetite, number of meals eaten at home or outside, alcohol and tobacco habits, physical activity, sleep habits and psychological variables. This survey considers a total of 27 variables for each participant.

A medical history was also registered at V0, including frequent medication intake, current diseases and family health history. This questionnaire asks about a series of medications (e.g. contraceptives or anti-inflammatory drugs) and diseases (e.g. hypertension or triglyceridemia) by default. Participants also described any other conditions that applied to them, resulting in fields with unstructured text. This questionnaire was manually curated in order to transform it into a structured data format. The 11 medications and eight diseases included in the questionnaire are encoded as binary variables, whereas those that were added by the participants are registered as qualitative variables in two additional tables. Two more binary variables encode those participants that added information about new medications and those participants that did not consume any. The same was done for diseases. Information about whether and when the participants had reached menopause was also collected when applicable.

Moreover, a general well-being measure was recorded during the manual-based intervention via the Spanish version of the Short Form-36 Health Survey (SF-36), a widely used generic health-related quality of life questionnaire  (41, 42). This survey consists of a total of 36 items (questions): 35 items that evaluate positive and negative health conditions, grouped into eight categories, and an additional item regarding the perceived change in general health status over the last year. Items are classified into the following scales:

- Physical functioning (10 items): this evaluates whether the participant can or cannot carry out physical activities (i.e. running, showering or going up the stairs) due to health problems.
- Role physical (four items): this measures problems during work or other daily tasks caused by physical health.
- Bodily pain (two items): this assesses whether the participant suffers from bodily pain that prevents them from performing daily tasks.
- General health (five items): participants are asked about their subjective perception of their own health.
- Vitality (four items): this provides a general measure of energy or fatigue.
- Social functioning (two items): whether physical or emotional problems hamper regular social activities.
- Role emotional (three items): if emotional problems negatively affect work or other daily tasks.
- Mental health (five items): this assesses anxiety, hopelessness, happiness or calm feelings.

Each item is given a numeric score and the summation of all relevant items is calculated for each scale. Then, a total score is calculated. Higher scores on this questionnaire are indicative of better overall health status. These variables were assessed twice for each participant, at the beginning and the end of the traditional-based intervention.

### Dataset 3: nutrition

During the 2-week manual data collection, participants recorded their nutritional habits using a food diary, recording all food and drinks consumed over 3 days. Participants completed this 3-day food diary twice: at the beginning and the end of the traditional-based intervention. Following guidelines from the European Food Safety Authority (43), this diary includes two weekdays and a weekend day or holiday from the week previous to the nutritionist visit. Participants were asked to register the mealtime and the weight of every food item where possible, using approximate measures (e.g. one cup, one teaspoon and a large plate). These registers are then processed using the DIAL commercial software (Alce Ingeniería, version 2.16, 2012) (44). This software is based on a database with the composition of >1000 food items in terms of energy, macro- and micronutrients. This is protected by copyright and can be accessed by purchasing the DIAL software license. As a result, >180 numeric variables are obtained, which can be summarized into the following:

- General parameters: the total calorie intake, as well as the number of servings of grains, fruits, vegetables, dairy products and protein foods, is calculated, as well as the Healthy Eating Index as defined by Kennedy *et al.* (45).
- Nutrient intake information: the Recommended Dietary Allowance (RDA), as well as the amount (weight) ingested and the percentage of the RDA that it represents, is given for a list of macro- and micronutrients.

In the same manner, participants were requested to capture images of all food and drinks consumed during the 2-week digital data collection. More precisely, they were required to upload all food images using the web platform described early.

### Dataset 4: biomarkers

Blood, urine and saliva samples were collected at the beginning and the end of the intervention (V1 and V3) in order to measure a series of biomarkers:

- Immune markers: tumor necrosis factor alpha, immunoglobulin levels (IgG, IgA, IgM and IgE) and immune cell populations, including lymphocytes, monocytes, segmented neutrophils, eosinophils and basophils.
- Biochemical parameters: triglycerides, high-density lipoprotein (HDL), low-density lipoprotein (LDL), total cholesterol, blood sugar levels, insulin, glycated hemoglobin, HOMA index, albumin, prealbumin, C reactive protein, adiponectin and leptin.
- Genetics: a series of single-nucleotide polymorphisms associated with metabolism, nutrition and immune system deterioration were measured.

Biochemical analyses of blood samples provided information from 39 variables, which were measured at two different time points: V1 (100 samples) and V3 (93 samples).

During the digital collection, participants were equipped with a CGM device that collected blood sugar levels on a 15-min basis, categorized into five different ranges: very high values (>250 mg/dl), high values (between 181 and 250 mg/dl), target range (70–180 mg/dl), low values (54–69 mg/dl) and very low values (<54 mg/dl). Additionally, the Glucose Management Indicator, which indicates the average AC1 level based on the average glucose level, glucose variability and coefficient of variation of all readings logged over 2 weeks (until the glucometer battery drained), were also computed.

### Dataset 5: gut microbiome

Apart from the biological samples described in the section ‘Dataset 4: biomarkers’, stool samples were also collected at V1 and V3 to characterize the gut microbiome composition and functionality via shotgun metagenomics and untargeted metabolomics. In total, 97 and 91 samples were collected at V1 and V3, respectively.

### Dataset 6: vital signs

Visits V0, V2 and V3 included clinical measurements of HR and blood pressure, stored in three variables: HR, systolic blood pressure and diastolic blood pressure. There are missing data points from two occasions in which participants attended the visit, but these measurements were not taken. In addition, the smartwatch utilized photoplethysmography to measure these parameters during the digital data collection. Photoplethysmography uses light to measure blood flow, allowing for continuous monitoring of vital signs in a non-invasive way. The following vital signs were captured using the wearable device:

- HR: it is recorded for each participant every 5 s as beats per minute (bpm). Additionally, the smartwatch provides HR zones, which are associated with physical activity and define how hard the exercise was based on four default zones: below zone 1 (no activity), zone 1 (low-intensity activity), zone 2 (medium-intensity activity) and zone 3 (high-intensity activity).
- ECG: participants performed a 30-s ECG test once a day, preferably at night when HR and stress levels were low. After completing the test, waveform samples with a 250-Hz frequency, the average HR and a result classification were stored. The test results included normal sinus rhythm (indicating normal heart rhythm), atrial fibrillation (indicating signs of atrial fibrillation) or an inconclusive result due to a high or low rate, or a poor reading.

### Dataset 7: physical activity

During the 2-week manual collection, participants completed the Spanish-translated version of the short last 7-day self-administered International Physical Activity Questionnaire (IPAQ) to record their physical activity (46). This questionnaire was completed twice by each participant: at the beginning and the end of the traditional-based intervention. This questionnaire consists of seven items that assess physical activity performed during leisure time, work, transport or domestic tasks lasting >10 min, as well as the time spent sitting. Activities are classified into three categories:

- Vigorous-intensity activity: these activities take a hard physical effort and require the participant to breathe harder than normal, such as heavy lifting or aerobics.
- Moderate-intensity activity: participants undertake a moderate physical effort and breathe harder than normal in this kind of activity (e.g. doubles tennis or lifting lighter weights). Walking is excluded from this category.
- Walking: this includes walks to or from work, transport, leisure walks, etc., that are >10 min.

These three categories are assessed by two items each: days per week and time spent each day. The seventh item asks participants about the amount of time they spend sitting every day. Then, each activity is weighted by its energy requirements in METs (multiples of the resting metabolic rate), estimating that vigorous-intensity activities require eight METs, moderate-intensity activities require four and walking requires four. These requirements are multiplied by the total minutes spent for each activity, yielding a score in MET-minutes for each kind of activity that serves as a quantitative measure of physical activity. A qualitative measure of physical activity based on three categories is also provided:

- Inactive: no physical activity or activity <600 MET-minutes per week.
- Minimally active: participants undertake either (i) 3 or more days of vigorous-intensity activity of at least 20 min per day; (ii) 5 or more days of moderate-intensity activity or walking during at least 30 min per day or (iii) 5 or more days with any combination of walking, moderate-intensity and vigorous-intensity activities, given that at least 600 MET-minutes per week are reached.
- Active: participants register either (i) 3 days of vigorous-intensity activity, reaching 1500 MET-minutes per week or (ii) 7 days of any combination of walking, moderate intensity and vigorous-intensity activities with a minimum of 3000 MET-minutes per week.

AI4FoodDB considers the answers to all seven items in numeric variables, including the MET values for each type of activity and their summation. The categorical classification for each participant and the observations recorded by nutritionists during each visit are also stored.

In addition to manually collected data, during the 2-week digital data collection, the smartwatch automatically detected each physical activity performed by the participant, including running, swimming, walking or cycling, among others. In this sense, HR data from participants during physical activity is also available in Dataset 6. We next provide the features collected by the FitBit smartwatch.

- Physical activity reports: this provides detailed reports of any physical activity. Calories, distance, duration, speed and additional specific information are included for each activity. For instance, swimming activity contains extra information about swim lengths, strokes or even lap duration in seconds.
- Active minutes: this parameter indicates the duration of physical activity based on four different activity levels: from sedentary and light activity to moderate and very active exercise. Furthermore, the device monitors and analyzes exercise intensity by identifying three active zones: fat burn zones related to moderate activity, peak zones related to hard activity and cardio burn zones related to vigorous activity. Each active zones is counted in minutes.
- Estimated oxygen consumption (VO_2_): measured in ml/kg, it represents an estimated maximum volume of oxygen that an individual can utilize during physical activity. The maximal oxygen consumption (VO_2_ max) values significantly vary for each individual due to several factors including age, gender, training and heredity, among others. In addition, filtered estimated maximum VO_2_ and computed errors are also stored.
- Additional physical activity data: other activity-related features such as calories burned per day, altitude, daily steps taken and distance covered per day (calculated from steps and strides) on a minute-by-minute basis.

### Dataset 8: sleep activity

Manual assessment of sleep habits was performed via the Oviedo Sleep Questionnaire (OSQ) (47) in two occasions: at the beginning and the end of the traditional-based intervention. The OSQ is a semi-structured interview that aids the diagnosis of insomnia and hypersomnia during the previous month based on the DSM-IV and ED-10 criteria, which has been validated in patients with depressive disorders. This questionnaire consists of 15 items, 13 of which are grouped into three different subscales:

- Subjective satisfaction with sleep: a single item, scored from 1 (very unsatisfied) to 7 (very satisfied).
- Insomnia: this scale is composed of nine items that contemplate difficulty with falling or remaining asleep, early awakenings or restorative sleep, among others.
- Hypersomnia: this scale contains three items that assess daytime sleepiness and its effects on daily tasks.

The two remaining items provide information about parasomnias and the use of sleep aids (e.g. medication and CPAP machine). Each item is scored on a 1–5 scale, except for the item related to subjective satisfaction with sleep. These items are stored as numeric variables, while the answers related to parasomnias and the use of help for sleeping, as well as clinical observations, are stored as text variables. Medical records for participants who use pharmacological drugs to help them sleep were reviewed and updated to also include this information.

Participants were instructed to wear the smartwatch during all sleep activities, primarily at night, for the digital data collection. The smartwatch automatically detects and records the different sleep stages whenever a participant starts to sleep, regardless of the time of the day. Similarly to Dataset 7 (physical activity), HR data from participants during sleep activity is also available in Dataset 6. The sleep-related features captured by the device are grouped into the following:

- Sleep quality: this feature is assessed using several scores computed each nightly sleep. The overall sleep score is calculated based on (i) the duration score, which monitors the total sleep time, (ii) the revitalization score, which measures how refreshed is the participant after waking up and is calculated from breathing disturbances and sleeping HR and (iii) the composition score, which considers the different nightly sleep stages. Other sleep parameters, including resting HR, duration of deep sleep stage and restlessness, which indicate the frequency of participant tossing and turning during the night, are also recorded.
- HR variability (HRV): this feature measures variations in the time range between heartbeats during the night’s sleep, and it is defined by five parameters. The parameter root mean square of the successive difference reflects vagal activity with normal sinus rhythm and is recorded every 5 min and globally for each nightly sleep. Additionally, coverage, low-frequency and high-frequency features are also recorded every 5 min. On the other hand, NREMHR (non–rapid eye movement HR), which measures the HR during light and deep sleep stages, and the entropy feature are captured nightly.
- Respiratory rate: measured in breaths per minute, the respiratory rate indicates the average breath count for each night’s sleep considering different sleep stages, including full sleep, deep sleep, light sleep and non -rapid eye movement (REM) sleep, along with their corresponding standard deviation (SD) and signal-to-noise ratio.
- Oxygen saturation (SpO_2_): this measures the average, lower bound and upper bound of SpO_2_ values for each night.
- Skin temperature: this measures the skin temperature in ^°^C taking samples every minute. It also records the wrist temperature throughout the day and additional relative sample features (e.g. baseline relative sample sum or sum of squares, among others).
- Additional sleep data: detailed reports of night sleep activities are also generated for each participant, including information about the start and end time of sleep, the duration of different sleep stages and time spent in bed, among other parameters.

### Dataset 9: emotional state

During the 2-week manual data collection, we also measured the psychological state of the participants using the short version of the Depression, Anxiety and Stress Scale (DASS-21) (48). As with the other validated questionnaires, the DASS-21 survey was completed twice by each participant. This self-reported questionnaire evaluates the emotional states of depression, anxiety and stress during the past week via three scales with 7 Likert items:

- Depression: this subscale assesses feelings such as hopelessness, lack of involvement or self-deprecation.
- Anxiety: these items evaluate subjective feelings and physical sensations such as a dry mouth, difficulty breathing or trembling.
- Stress: this scale rates feelings of agitation, irritability or impatience, as well as nervous arousal.

All items are rated on a scale from 0, if they did not apply to the participant at all, to 3, if it applied to them very much or most of the time. A score for each scale is calculated by summing all the related items and these scores are later added to obtain a general score.

On the other hand, during the 2-week digital data collection, the emotional state was measured using the FitBit measurements of stress levels produced by the daily routine. The smartwatch considers some lifestyle factors such as sleep, exercise and mental well-being.

- Stress score: it comprises a daily stress management score derived from 10 factors grouped into three categories: sleep patterns, responsiveness that are related to heart-rate data and EDA activity, and exertion balance computed from physical activity. The final score ranges from 1 to 100, meaning a higher score fewer physical signs of stress.
- EDA sessions: the device records HR- and EDA-related features such as the skin conductance levels (SCLs) for each test. In this case, differences in the SCL indicate trends in the body’s response to stress and are directly associated with the sympathetic nervous system.

### Dataset 10: additional information

Cognitive impairment was measured once (V2) using the Spanish version of the Mini-Mental State Examination (MMSE) by Folstein *et al.*  (49, 50), which includes a series of five items with a maximum punctuation of 35 points. Scores <3 are indicative of cognitive impairment. A total of 98 participants underwent the examination.

Participants also completed a survey regarding COVID-19 severity and persistence. Vaccination state, as well as the severity and duration of COVID-19 symptoms for those participants that had suffered from the disease, was recorded. The information about vaccination status and the timing of participants’ COVID-19 diagnoses is encoded in 15 variables. Additionally, participants reported information about the symptoms they suffered. Thus, 23 different symptoms are included in the dataset, with five additional variables to further characterize them. A total of 99 participants completed the survey. This information was collected at a single visit for each participant.

A System Usability Scale (SUS) consisting of 10 different items was handed out to the participants at the end of the intervention in order to evaluate adherence to digital data collection devices. This survey was answered by 96 participants at a single time point, corresponding to the last visit of the digital-based intervention.

### Problems encountered during the acquisition

A list of challenges has been gathered during the AI4FoodDB acquisition, including those related to manual and digital data, and the utilization of user-friendly technologies.

- Data collection based on printed questionnaires implies the manual entry of the collected data to a computer. This process is time-consuming and mistakes can be made, thus requiring a subsequent step of data curation. Here, all the questionnaires have been reviewed and curated to solve inconsistencies, identify missing data points and convert all the information into a structured data format when necessary.
- Besides the mentioned limitations related to manual data entry into a database, both manual and digital-based data collection approaches can be biased. Dietary assessment based on food diaries is highly dependent on the subject’s perception of portions, as well as when uploading food images to the web platform.
- Given the complexity of collecting continuous signals from multiple devices and relying on participants to synchronize their data, minor information was missed or not properly stored. Specifically, regarding the smartwatch, some tests related to Datasets 6, 8 and 9 were not registered in those participants who took off the smartwatch while sleeping or did not perform them correctly (~15% of the total data). For the glucose device, ~10% of the total data were lost due to a lack of synchronization (from >29 000 h of data). In addition, some meals were not uploaded by the participants to the food logging web platform (~23% of the total meals). As can be seen from the figures, this minor lack of information does not affect the correct development of the research project and it is in line with real-life acquisitions.
- There is no standardization of the format of files from digital devices. Therefore, the preprocessing of these files requires a complex and time-consuming task.
- Recent digital technologies do not seem to be user-friendly for elderly people yet. Some of the main challenges encountered by a few participants, particularly the elderly, include NFC technology, synchronization between wearable devices and smartphones and even the use of web platforms to upload images.
- Around 7% of the glucose devices stopped working after a few days, requiring the devices to be replaced.

## Case study: participant follow-up

**Figure 3.1:**
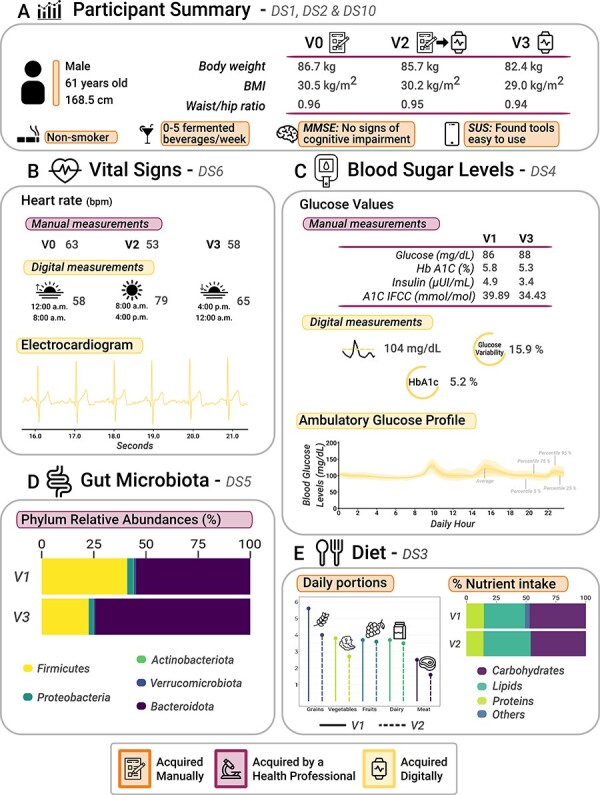
Graphical analysis of participant data (a 61-year-old male) collected from AI4FoodDB during the clinical intervention. (**A**) Participant summary, which includes anthropometric measurements from the three visits and additional information (extracted from Datasets 1, 2 and 10). (**B**) Vital signs associated with manual and digital measurements (Dataset 6). (**C**) Blood sugar levels, which include manual and digital measurements (Dataset 4). (**D**) Gut microbiota: relative abundances of the five most abundant phyla at V1 and V3 (Dataset 5). (**E**) Diet (Dataset 3).

**Figure 3.2:**
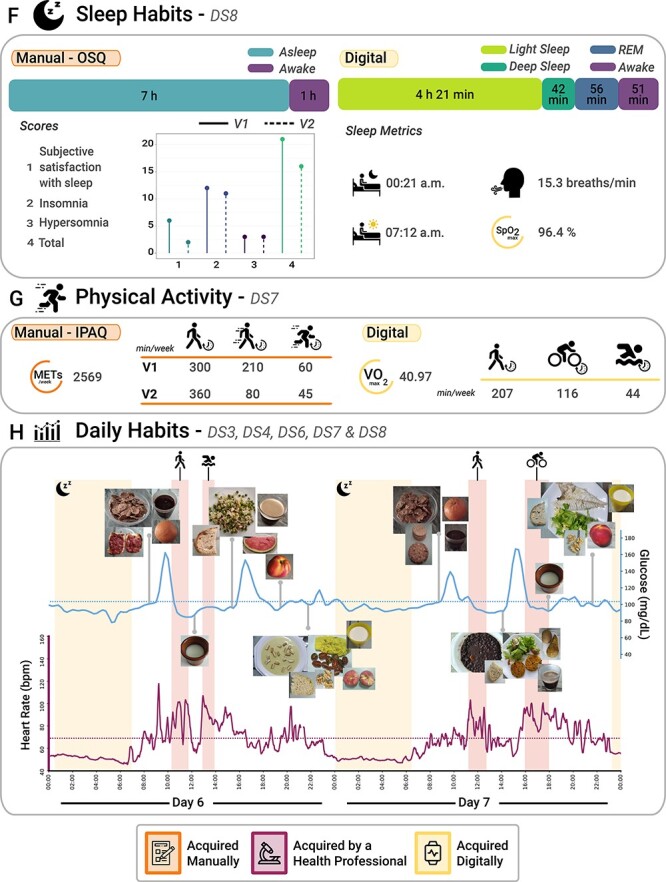
Continuing from Figure 3.1. (**F**) Sleep habits from both subjective (OSQ) and objective (wearable devices) perspectives (extracted from Dataset 8). (**G**) Physical activity from both subjective (IPAQ) and objective (wearable devices) perspectives (Dataset 7). (**H**) Daily habits, which comprise a 2-day report that includes blood glucose levels, HR, night sleep activity, physical activity and images of the different foods consumed during these 2 days (Datasets 3, 4, 6, 7 and 8).

AI4FoodDB can be used to retrieve information regarding different lifestyle, health and nutritional variables from each participant. To provide an example, Figure 3.1 and 3.2 summarize the data collected for a single participant, comparing the follow-up that was carried out with digital and traditional methods for each of the datasets. We also compare how different variables were assessed during different parts of the intervention, providing an example of how these data can be integrated to monitor lifestyle habits.

The selected participant is a 61-year-old male that started the intervention with a BMI of 30.5 kg/m^2^, finishing with a BMI of 29.0 kg/m^2^ and a body weight reduction of 4.3 kg (4.96% of his body weight), as depicted in Figure 3.1A. This information is included in Dataset 1. For a general idea of the participants’ lifestyle and health state, we can access the lifestyle survey (included in Dataset 2), which reveals that this participant does not smoke and has a moderate alcohol intake of up to five fermented beverages a week. Other questionnaires can also provide relevant information: for instance, the MMSE revealed no signs of cognitive impairment. Moreover, according to the SUS survey, he did not encounter any major issues when using digital tools. Both questionnaires are included in Dataset 10. This participant was randomized into Group 1, meaning that he was assessed via traditional methods during the first 2 weeks (V1 and V2) and via digital methods during the following 2 weeks (V2 and V3).

Figure 3.1B shows how vital signs (Dataset 6) were assessed during the 2-week digital and manual interventions. A static measurement can be retrieved by accessing the blood pressure and HR measured by nutritionists during the different visits (V0, V2 and V3). Additionally, the digital intervention allows for the continuous measurement of HRs and the analysis of how they relate to various activities (refer to Figure 3.2H). Higher HRs are directly associated with physical activities or movements, certain moments during the meal or after waking up. In contrast, lower HRs are observed during sleep or rest periods, or even after eating, among other activities. Overall, the participant exhibited lower HRs during mornings and nights, with an average HR of 58 bpm from 0 to 8 h and a slightly higher average HR of 65 bpm from 16 to 24 h. In contrast, the participant’s average HR during the day (from 8 to 16 h) was 79 bpm. An ECG is displayed in the figure for completeness.

In addition to vital signs, several biomarkers (Dataset 4) were measured via biological sample collection at the beginning and the end of the intervention (V1 and V3). Moreover, glucose levels were continuously monitored during the digital intervention (V2 and V3). Thus, we have static measurements of fasting blood glucose, insulin levels and glycated hemoglobin levels for visits V1 and V3, which are accompanied by continuous monitoring of blood glucose levels from V2 to V3 as depicted in Figure 3.1C. A blood glucose level summary over the 2-week digital intervention is also included: glucose levels averaged a value of 104 mg/dl, a 5.8% of the hemoglobin A1c (HbA1c) level and a 15.9% glucose variability. As an example, the Ambulatory Glucose Profile of a 24-h interval is also provided. Moreover, Figure 3.2H shows this profile in combination with other information such as dietary intake (Dataset 3), sleep (Dataset 8) and physical activity (Dataset 7). High glucose peaks are directly related to when the participant ate the different meals. High glycemic index foods such as rice, bread, breakfast cereals or sugary foods mean a higher blood glucose level reflected in the participant, while low-medium glycemic index foods (e.g. fruits, vegetables or fish) produce a lower glucose peak. Finally, GM composition is also proposed to be relevant in NCDs such as obesity. Here, the relative abundances of the different microbial species identified can be retrieved (Dataset 5). Figure 3.1D shows a summary of the top phyla that were found at the beginning and the end of the nutritional intervention (V1 and V3).

Dietary habits were documented through photographs and food diaries (Dataset 3). Figure 3.1E provides a summary of the macronutrients and food-type data via food diaries. The number of portions of grains, vegetables, fruits, dairy products and meat is provided on the left. On the right, the amount of macronutrients consumed is represented as the percentage over the total energy value. Conversely, Figure 3.2H shows a 2-day eating pattern captured through food images uploaded by the participant to the web platform. We include the precise moment when the images were taken to compare them with blood glucose levels and the HR signal data. Both approaches agree on a high intake of vegetables, fruits and grains, which are present in every meal. The traditional approach allowed for nutrient intake calculations, while the digital approach yielded valuable visual data.

In this study, we demonstrate how AI4FoodDB can be used to retrieve general lifestyle information as well as data related to different biomarkers. Regarding lifestyle information, we can track sleep habits (Dataset 8), physical activity (Dataset 7) and dietary intake (Dataset 3). Figure 3.2F shows the differences in sleep habits information for the participant in question. These data sources report different types of information. While we can compare basic parameters such as hours of sleep or awakeness, more research is required to analyze how the different OSQ scores might relate to the sleep stages detected by the smartwatch. During the manual intervention, the participant reported spending 8 h of sleep, out of which seven were spent sleeping. The OSQ also provides different scales to assess sleep quality, as shown in the figure. During the digital intervention, the participant slept an average of 6 h and 51 min, from which 4 h and 21 min was in the light sleep stage, 42 min in the deep sleep stage, 56 min in the REM stage and ~51 min awake (sometimes unnoticed by the participant). In addition, the average bedtime was ~00:21 a.m. and the average wake-up time was ~07:12 a.m. Notably, both blood glucose levels and HR signal were typically low during sleep, as shown in Figure 3.2H.

Lastly, the information related to physical activity retrieved from the traditional collection is the minutes spent carrying out physical activities of different intensities (i.e. walking, moderate-intensity and vigorous-intensity activities), along with an estimate of their energy requirements (Figure 3.2G). Digital data also provide information on the specific activity performed: the participant went for a walk ~207 min per week, rode a bicycle ~116 min per week and swam ~44 min per week. Moreover, the overall VO_2_ calculated from all activities was 40.97. Figure 3.2H shows four physical activities performed by the participant during the 2-day habit report, including two walks, one bicycle road and one swim. Data from both manual and digital interventions reveal that this participant is highly active.

This case study shows the potential of digital data collection methods in e-Health, as they enable continuous and in-depth monitoring of lifestyle habits compared to manual data collection approaches. The reduction of body weight and waist/hip ratio after the intervention is accompanied by lifestyle changes such as lower meat consumption, reduced glycated hemoglobin levels and maintaining an active lifestyle with high levels of physical activity.

## Conclusions

Diet-related chronic diseases represent today many challenges in the nutrition and health-care system. Personalized dietary recommendations result beneficial for both clinicians and patients, but existing tools are still far from optimal. Emerging technologies such as e-Health and wearable devices offer a promising and cost-effective means of characterizing each patient. However, the implementation of such approaches in clinical practice requires appropriate techniques for data processing and interpretation. To this end, we have monitored 100 obese and overweight participants with different methods, collecting diverse data that we have gathered into AI4FoodDB.

The use of e-Health strategies in nutritional and clinical interventions has been evaluated elsewhere, showing positive effects in NCDs such as T2D, obesity or metabolic syndrome (51–54). However, the need to compare these approaches to those currently used in health systems has been pointed out (52), and it seems that the use of digital technologies in interventional studies might be effective when combined with in-person consultations, rather than as a substitute (51). Lastly, none of these reviews contemplates the use of pictures for dietary tracking together with mobile-based resources. Therefore, we are confident that AI4FoodDB is a unique resource in this field, as (i) a crossover design was followed, allowing to compare the use of traditional and digital methods, (ii) participants attended nutritionist consultations every 2 weeks, warranting close follow-up by health-care providers and (iii) very diverse data collection techniques are used, resulting in a thorough and integrative characterization of each participant.

AI4FoodDB is completely aligned with the efforts carried out by the food and nutrition community to create resources and infrastructures that support the development of the personalized nutrition, like those from the ELIXIR F&N Community (5). The European Open Science Cloud (https://eosc-portal.eu/) and the Food Nutrition and Security Cloud (FNS-Cloud, https://www.fns-cloud.eu/) have stated the fragmentation of the datasets, the poor metadata annotation and the lack of FAIRness of the data resources in the nutrition field. Moreover, improved and new databases are required to overcome the different challenges for the deployment of personalized nutrition, as described by the American Nutrition Association (55) or the ELIXIR F&N Community (5). AI4FoodDB is an open and FAIR database with very complete and rich information from different sources, like clinical assessments, biomarkers, omics data or biosensors derived from advanced technologies. We believe that the information provided in AI4FoodDB will be very valuable for the development of the personalized nutrition field, supporting the activities of the ELIXIR F&N Community (5) or those to create nutrition infrastructures, like PI HDHL INTIMIC knowledge platform (https://www.healthydietforhealthylife.eu/), FNS-Cloud or the ESFRI research infrastructure: Food, Nutrition and Health RI (https://fnhri.eu/).

Our goals include the generation of AI techniques to analyze this large collection of data, providing the research community with algorithms that allow them to analyze multimodal datasets. Moreover, the large amount of information provided by AI4FoodDB will open new research lines in the near future, enhancing our comprehension of individuals’ demands and enabling us to combat the most prevalent diseases in contemporary society. The holistic human body map obtained here will merge different areas comprising health informatics, nutrition, biology or AI, among others.

## Data Availability

AI4FoodDB is available at https://github.com/AI4Food/AI4FoodDB.
